# Deltoid ligament injuries: A review of the anatomy, diagnosis and treatments

**DOI:** 10.1002/ksa.12274

**Published:** 2024-05-26

**Authors:** Jacob Koris, James D. F. Calder, Miki Dalmau‐Pastor, Miguel A. Fernandez, Arul Ramasamy

**Affiliations:** ^1^ Trauma & Orthopaedic Specialty Registrar, John Radcliffe Hospital Oxford UK; ^2^ Department of Bioengineering Imperial College London London UK; ^3^ Fortius Clinic London UK; ^4^ Human Anatomy and Embryology Unit, Department of Pathology and Experimental Therapeutics, School of Medicine and Health Sciences University of Barcelona Barcelona Spain; ^5^ MIFAS by GRECMIP (Minimally Invasive Foot and Ankle Society) Merignac France; ^6^ Warwick Medical School University of Warwick Coventry UK; ^7^ Academic Department of Military Trauma and Orthopaedics Royal Centre for Defence Medicine, Edgbaston Birmingham UK

**Keywords:** ankle instability, deltoid ligament, medial ankle injury, soft tissue ankle

## Abstract

**Purpose:**

Ankle sprains remain the most common soft tissue injury presenting to Emergency Departments. Recently, there has been increased awareness and reporting of deltoid ligament injuries in association with injuries to the lateral ligament complex as well as with fibula fractures. This article reviews the currently available literature on the anatomy of the deltoid ligament, clinical and radiological diagnosis of injuries to the deltoid ligament and treatment recommendations.

**Methods:**

A literature review was conducted for keywords associated with deltoid ligament injuries. MEDLINE, PubMed and Embase databases were utilised for this search. Articles were included if involving an adult population, were English‐language, were related to deltoid ligament injuries (with or without associated injuries) and reported on patho‐anatomy, clinical or radiological diagnosis or treatment methods.

**Results:**

A total of 93 articles were assessed for relevance from the database search, and 47 were included after the removal of irrelevant articles and duplicates. Several studies reported on the clinical findings of deltoid ligament injury, as well as the radiographic analysis. Arthroscopy was considered the gold standard of diagnosis, with authors reporting on the potential benefit of performing arthroscopic repair or reconstruction at the same time. There were no studies that provided a system for the classification of deltoid ligament injury or larger studies of treatment pathways. Long‐term studies of the incidence of instability in deltoid ligament injuries were not available.

**Conclusion:**

There is limited evidence available regarding deltoid ligament injuries, particularly in terms of treatment options, either in isolation or with concomitant injuries. Long‐term follow‐up studies are needed to obtain more accurate data on the number of complications.

**Level of Evidence:**

Level IV.

**Abbreviations**
AAOS‐FAM: American Academy of Orthopedic Surgeons Lower Limb Outcomes Assessment: Foot and Ankle Module
AOFAS: American Orthopedic Foot & Ankle Society
AP: antero‐posterior
ATT: anterior tibiotalar ligament
ATTN: anterior tibiotalonavicular ligament
dPTT: deep posterior tibiotalar ligament
ITT: intermediate tibiotalar ligament
MCS: medial clear space
MRI: magnetic resonance imaging
NFL: National Football League
NICE: National Institute for Clinical Excellence
ORIF: open reduction internal fixation
SF‐36: 36 Item Short Form Survey
sPTT: superficial posterior tibiotalar ligament
TCC: tibiotalocalcaneal ligament
VAS: Visual Acuity Scale

## INTRODUCTION

Ankle sprains and injuries are common, with an incidence of 52.7–60.9 per 10,000 in the UK population [7]. An estimated prevalence of 302,000 new ankle sprains present to Accident & Emergency in the United Kingdom each year, with 42,000 of these being classified as ‘severe’ [7]. The majority of ankle sprains involve injuries to the lateral ligament complex, with some authors estimating only 5% of ankle sprains affect the deltoid ligament [79]. However, there is increasing recognition that medial ligamentous injuries may be more frequently associated with ankle sprains [72] and fractures [28] than previously reported. Medial ankle instability has been associated with eversion‐type injuries to the ankle, and 3%–4% of all ankle ligament injuries are isolated to the deltoid ligament [72].

Although the precise anatomy of the deltoid has become better described over time, both the incidence and significance of partial and complete tears to the superficial and deep components are not well understood. Therefore, there are inconsistent recommendations in the literature as to the management and need for surgical repair following deltoid ligament injury.

The purpose of this review is to critically assess the literature with regard to the patho‐anatomy of deltoid ligament injuries, the clinical findings, diagnostic imaging and the rationale for treatment strategies.

## MATERIALS AND METHODS

The National Institute for Clinical Excellence (NICE) Healthcare Databases Advanced Search [60] was used to simultaneously search EMBASE, PubMed and Medline with search terms, ‘deltoid ligament injury’, or ‘deltoid ligament tear’, or ‘deltoid ligament rupture’ between 1980—the present.

Inclusion criteria:

1. Clinical, cadaveric, anatomical and biomechanical studies of the pathology (including patho‐anatomy), clinical evaluation, diagnostic imaging or treatment of acute or chronic deltoid ligament injuries with or without underlying fracture;
2. An adult (more than 18 years old) population;
3. English language articles.

After the removal of duplicates, all articles were screened and the inclusion/exclusion criteria were applied.

Once articles were identified, authors (J. K., A. R.) extracted the relevant data for inclusion in the review. Manuscript data such as title, authors, level of evidence, as well as study‐specific data such as diagnostic criteria, examination findings, diagnostic imaging findings, comparisons and outcomes were included.

## RESULTS

This provided a total of 93 articles. All articles had their titles assessed for relevance, and 26 were excluded on that basis. There were 20 duplicate articles that were removed. The remainder of the articles (47) were included in the literature review.

### Anatomy & function

#### Anatomy

The accepted description of the deltoid ligament divides it into six fascicles formed into two planes (superficial and deep) [57]. Four fascicles are described within the superficial layer: tibionavicular and tibiospring, present in every case; and superficial tibiotalar and tibiocalcaneal, nonconstant fascicles. In the deep layer, two fascicles: posterior tibiotalar, always present; and deep anterior tibiotalar, nonconstant.

However, when reviewing all the literature published on the anatomy of the deltoid ligament, this description has mutated, as up to 16 fascicles have been described and reported in the deltoid ligament. While some authors have stuck to those six fascicles [5, 8, 20, 95], Cromeens et al. added to that description the ‘tibiocalcaneonavicular’, the ‘superficial posterior tibiotalar’, the ‘deep posterior tibiotalar’ and the ‘inferoplantar longitudinal ligaments’ [10]. The ‘band deep to tibiocalcaneal ligament’ and ‘band posterior to sustentaculum tali’ were added by Panchani et al. [65]. Zamperetti et al found one accessory bundle, the ‘deep intermedial tibiotalar ligament’ [98]. Some of these fascicles could be the one Rasmussen reported as the ‘intermediate tibiotalar ligament’ in 1983 [70], as they all seem to lie deep in the tibiocalcaneal fascicle; however, this was not discussed in the articles. Ismail et al. [34] recently published a study adding the ‘tibiotalocalcaneal ligament’ as a constant fascicle, and the ‘anterior tibiotalonavicular ligament’, present in 60% of cases, together with four of the formerly mentioned fascicles.

Furthermore, a 2022 study of 30 embalmed cadaveric ankles, studied by gross examination, micro‐dissection and light microscopy [70] found six ligamentous bands. Two superficial variants were found: The tibiotalocalcaneal ligament (TTC) and the superficial posterior tibiotalar ligament (sPTT). Four deep variants were described: the anterior tibiotalar ligament (ATT), the anterior tibiotalonavicular ligament (ATTN), the intermediate tibiotalar ligament (ITT) and the deep posterior tibiotalar ligament (dPTT). Of these, the TTC was identified in all specimens, whereas the five additional bands were variable [34].

**Figure 1:**
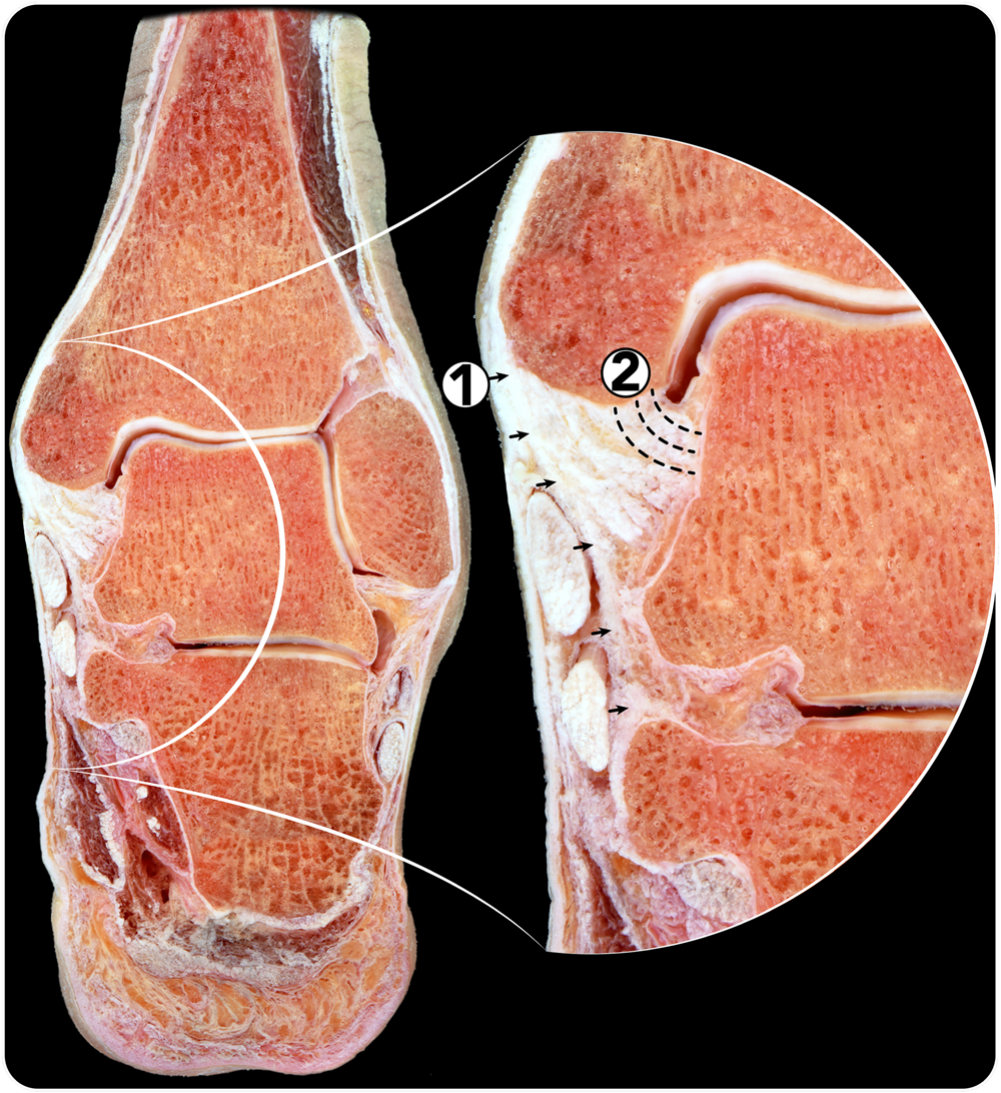
Frontal cross‐section of a specimen showing the distribution of the deltoid ligament in two planes. 1. Superficial deltoid. 2. Deep deltoid.

**Figure 2:**
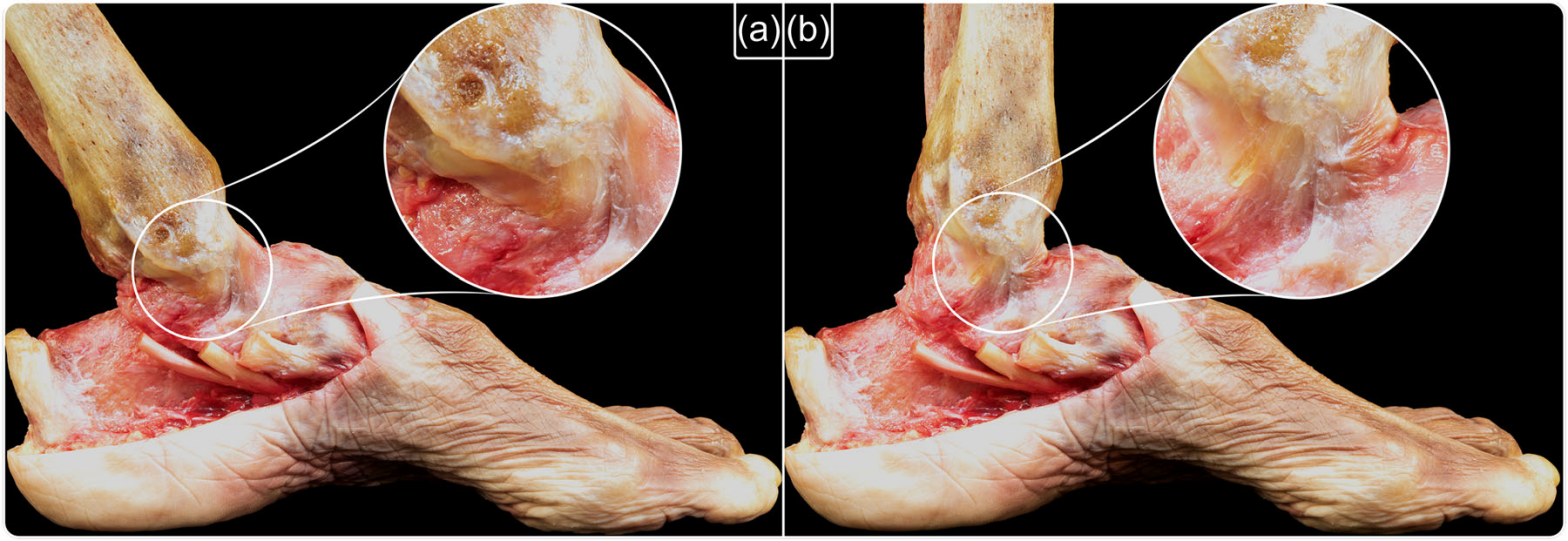
Medial view of an osteoarticular dissection showing the tension of deltoid ligament fibres. (a) Ankle plantarflexion (anterior deltoid fibres are tense), (b) ankle dorsiflexion (posterior deltoid fibres are tense).

Finally, it appears that regardless of the number and frequency of fascicles, the only real consensus in the literature is that the deltoid ligament is organised in two planes, one superficial and one deep (Figure 1). On the other hand, due to its triangular morphology, expanding distally, the anterior part of the deltoid ligament is tense in plantarflexion, while the posterior part is tense in dorsiflexion (Figure 2).

This review of anatomical papers published to date is probably a good example of how confusing the current description of the deltoid ligament is, and the problems clinicians face when reporting injuries of this structure. This becomes a problem also for repair and reconstruction surgical procedures, as the lack of a detailed description creates uncertainty regarding which part of the ligament is being repaired/reconstructed and makes the comparison between studies difficult.

### Patho‐anatomy

The primary mechanism of injury to the deltoid ligament is the eversion of the ankle, with or without external rotation [64]. This typically involves landing on uneven ground on a pronated foot [25]. Deltoid ligament injuries are also found in high‐energy trauma patients, often associated with complex fracture or dislocation patterns [17]. Supination and external rotation injuries have also been shown to lead to deltoid ligament injuries due to the excessive external rotation of the talus, often with associated injuries to the syndesmosis or tibiofibular ligaments [50].

A Magnetic Resonance Imaging study of injured ankles with medial instability found that the most common location of injury was to the superficial layer of the deltoid ligament at the tibial periosteal attachment [56].

Ankle fractures almost always involve injury to the surrounding ligaments including the lateral ligament complex [42, 43] and the syndesmosis [9], and it has been estimated that up to 40% of ankle fractures will have concomitant injury to the deltoid ligament [26, 45]. An observational study of patients with ankle fractures requiring surgery for instability found that deltoid ligament injury was a more common finding than medial malleolar fractures (56% vs. 44%), and those with deltoid ligament injury tended to be younger.

An arthroscopic study using the Lauge‐Hanson classification of ankle fractures determined that Supination‐External Rotation Type IV and Pronation‐External Rotation Type IV injuries resulted in deltoid rupture in 86% of cases [78]. This finding is supported by a biomechanical study of deep deltoid ligament injuries contributing to rotational instability of the ankle joint published in 2021 [49].

A recent biomechanical study showed that the superficial and deep deltoid have equally important roles in the stability of Supination‐External Rotation IV ankle fractures and challenges the idea that injury to the deep deltoid must have occurred if instability is present [23].

### Clinical evaluation

#### History

Patients may present to the hospital acutely at the time of injury, subacutely with a delayed presentation or chronically, once complications occur. Patients may report medial ankle pain, with a tearing sensation at the time of injury [54].

More chronic cases may present with persisting medial ankle pain (particularly in the gutter), with ongoing swelling and tenderness [29, 30, 54]. The sensation of the ankle ‘giving‐way’, particularly of the medial aspect of the ankle and on activity may be reported in subacute cases [25].

#### Physical examination

Tenderness in the medial gutter, distal to the tip of the medial malleolus is the most common finding and in a case series of 52 patients, Hintermann et al. found that 100% of patients presenting with instability also have tenderness in the medial gutter [30, 62]. Although the presence of medial gutter pain is highly sensitive for deltoid ligament injury, it is not specific in acute ankle injuries, with DeAngelis et al. demonstrating no correlation between medial ankle tenderness and evidence of deep deltoid ligament insufficiency on external stress testing [13].

Egot et al. investigated the findings of medial tenderness, swelling and medial bruising alone, and reported sensitivities from 20% to 56% and specificities from 71% to 97% [15].

Stress tests can be performed in order to further evaluate the deltoid ligament, including the talar tilt test, valgus stress test and external rotation test [13]. Anterior and medial subluxation on the anterior draw test is a sign of deltoid ligament injury [25].

It is important to recognise that these tests are all considered inadequate for the evaluation of a deltoid ligament injury without further imaging [14].

A valgus heel deformity is a sign of a chronic deltoid ligament injury with associated posterior tibial tendon dysfunction (associated or not with flat‐foot deformity) [30].

Ruiz in 2021 described a pathognomonic clinical finding of a valgus hindfoot with pronation deformity that resolves with activation of the posterior tibial muscle (or going into the tip‐toe position) as diagnostic for medial ankle instability [74].

#### Classification systems

Classification systems are useful in order to communicate diagnosis, predict prognosis and determine treatment protocols. There are two classification systems that can be used for deltoid ligament injuries: the first is a general system used to describe ligament injuries and the second is specific for the deltoid ligament.

General classification of ankle sprains—Classification based on the degree of tear of ligaments with a description of signs and symptoms (see Table 1) [64]. It should be noted that the superficial and deep layers may have different grades of injuries and should be commented on separately.

- Type 1—Partial tear of a ligament.
- Type 2—Incomplete tear with moderate functional impairment.
- Type 3—Complete tear and loss of integrity of a lesion.

**Table 1:** Table showing grading system of ligament tears with associated signs & symptoms.

Grade	Signs & symptoms
I: Partial tear of a ligament	Mild tenderness and swelling
	Slight or no functional loss
	No mechanical instability
II: Incomplete tear with moderate functional impairment	Moderate pain and swelling
	Mild to moderate ecchymosis
	Tenderness over involved structures
	Some loss of motion and function
	Mild to moderate instability
III: Complete tear and loss of integrity of a lesion	Severe swelling
	Severe ecchymosis
	Loss of function and motion
	Mechanical instability

Hintermann Classification of Deltoid Ligament Injuries—The Hintermann Classification is based on the location of the tear and the ligament elements involved (see Table 2) [25].

- Type 1—Proximal Tear or Avulsion; involving the Tibionavicular +/– Tibiospring Ligament.
- Type 2—Intermediate: Deep component remains attached distally and superficial component remains attached proximally; involving the Tibionavicular +/– Tibiospring Ligament.
- Type 3—Distal Tear; involving the Tibionavicular +/– Spring Ligament.

**Table 2:** Table showing the Hintermann Classification of Deltoid Ligament Injuries with lesion type, location of the tear within the deltoid ligament, the ligament involved and the incidence.

Lesion Type	Location	Ligament Involved	Incidence (%)
I	Proximal tear or avulsion	Tibionavicular; Tibiospring	72
II	Intermediate: Deep component remains attached distally; superficial component remains attached proximally	Tibionavicular; Tibiospring	9
III	Distal Tear	Tibionavicular; Spring	19

### Imaging

#### Radiographs

In acute injuries, the patient is often unable to weight‐bear. Standard antero‐posterior (AP), lateral and mortice view radiographs are performed to exclude concomitant fractures around the ankle. If any proximal fibular pain is present, radiographs of the knee must be performed to exclude a Maisonneuve‐type injury.

AP radiograph may reveal a medial malleolar ‘fleck sign’, which Nwosu et al. felt represented an indication of an unstable supination‐external rotation ligamentous injury to the ankle; it could influence patient treatment, as well as the need for further investigations [63]. This retrospective observational study included 166 ligamentous supination external‐rotation ankle injuries assessing for evidence of medial clear space (MCS) widening in static and dynamic conditions. They found that 94% of patients with a ‘fleck sign’ [15 of 16 patients) had an increased MCS, and therefore evidence of instability.

Therefore, if the history or examination is suggestive for deltoid ligament injury, further imaging is indicated [44].

### Stress radiographs

Stress radiographs can either be performed in the outpatient setting, or intraoperatively with fluoroscopy. Stress radiographs are performed looking for the talus to tilt into a valgus position.

Tests include the gravity view, manual valgus and external rotation stress view. Findings on stress view indicating an isolated deltoid ligament injury is a valgus talus tilt without any talus shift [46]. It is generally felt that 4–6 mm of widening is indicative of a positive result for deltoid ligament laxity [14, 55, 66, 69]. An arthroscopic study by Schuberth et al. demonstrates that using an MCS of 3 mm had a false positive rate of 88.5%, which fell to 26.8% for an MCS of 5 mm and 7.7% with an MCS of 6 mm.

Van Leeuwen et al. have demonstrated that gravity stress views provided significantly larger MCS measurements compared with simple mortise views for diagnosing deltoid ligament injuries [88]. Gravity stress views were also superior in sensitivity, specificity and negative predictive value when compared with simple mortise views.

A prospective case–control study by Jeong et al. compared likelihood ratios for gravity stress views and external rotation stress tests. Their study of 37 patients suggested that while gravity stress views are somewhat better than clinical findings in attaining a positive diagnosis (LR = 5.7 vs. 2.0), the findings need to correlate with a positive MRI result in order to be as good as external stress views for diagnosing deltoid ligaments injuries [35]. These findings echo that of Gill et al. who compared the MCS in patients with Supination‐External Rotation II and IV injuries for both gravity and manual stress radiographs. They found that in unstable Supination‐External Rotation IV injuries, manual stress radiographs provided significantly higher MCS measurements than gravity alone (5.2 vs. 5.0 mm, *p* < 0.05), but both tests were significantly different to those with stable Supination‐External Rotation II injuries (4.2 vs. 4.3 mm respectively) [19]. This study of 25 patients suggests that gravity stress views are equivalent to manual stress radiographs in determining deltoid ligament injury.

A further study of 29 patients by Schock et al. compared the efficacy of the gravity and external stress view radiographs [77]. No significant difference in MCS was found between the two methods (6.1 mm vs. 5.8 mm, n.s.). This study included a comparison of changes in patient‐reported pain scores and found that the gravity‐assisted view was significantly more comfortable for the patient (0.6 vs. 2.9, *p* < 0.0001).

When performing intraoperative stress tests, it is important to conduct the manoeuvres with the ankle in neutral [14], achieve the appropriate amount of external rotation [44] and perform the test with the ankle in both varus tilt [16]. It has been well demonstrated that foot position and the location at which the MCS is measured can have a large effect on the measurement; this must be taken into consideration, and may make fluoroscopic assessment easier and with less overall radiation exposure to the patient [4].

In a cadaveric study of six fresh ankles, Park et al. investigated the effect that positioning had on MCA and found that an MCS of 5 mm taken in dorsiflexion external rotation provided a sensitivity, specificity and negative and positive predictive value of 100%. Furthermore, of the positions tested (planter flexion, neutral, dorsi flexion), external rotation in dorsiflexion was most predictive of deep deltoid ligament insufficiency [66].

### Ultrasound

Ultrasound assessment is an effective method of assessing for complete tears of the deltoid, but may miss more complex partial tears, and has limited function in the assessment of concomitant ligamentous or bony injuries [67]. In some centres, where access to other imaging modalities is limited, ultrasound may represent a more time‐sensitive method of diagnosis [58]. A study of 100 patients found that ultrasound was superior to simple radiographs, identifying deltoid ligament injuries in 39% of 100 patients with ankle injuries [1]. The main benefit of ultrasound is that dynamic testing of the deltoid ligament can be performed [75]. Displacement of the medial structures during an eversion stress test is diagnostic of a deltoid ligament injury [67].

A study comparing the sensitivity and specificity of ultrasound and gravity stress test found a higher sensitivity (100% vs. 97%) but lower specificity (90% vs. 100%) with ultrasound [32].

### Magnetic resonance imaging (MRI)

MRI remains the gold standard for assessing soft tissue pathology. Axial MRI with local gradient represents the best imaging modality allowing for assessment of both the superficial and deep aspects of the deltoid ligament, as well as the individual bands of the superficial layer. This allows for a full assessment of the deltoid ligament in injured patients [59]. Given the fact that deltoid injuries often occur with associated injuries [26], MRI can also assess for lateral ligament complex injuries, syndesmotic injuries, osteochondral injuries and fractures [36, 39].

A study assessed all patients being operated on for Supination‐External Rotation Type IV ankle fractures, with intraoperative assessment of the deltoid ligament as a gold standard. They determined that patients with an MCS of 5 mm on weight‐bearing radiographs were diagnostic. Those with an MCS of less than 5 mm should have a further assessment with MRI due to its increased accuracy and decreased false positive rate [92]. Interestingly, MRI studies have demonstrated that in patients with fibular fractures, associated syndesmotic and/or deltoid injuries were identified in the majority of cases [24]. However, MRI has not been shown to assist in determining whether patients require reconstruction [61].

### Arthroscopy

Assessment of the ankle with arthroscopy can be useful in order to assess for deltoid ligament injuries, osteochondral injuries and instability with stress testing. Described stress tests include axial traction, talar anterior draw, valgus stress and varus stress testing [51]. A cadaveric study of 20 fresh frozen ankles found that adequate ankle dorsiflexion allowed for full visualisation of the medial and lateral ligaments in all 20 ankles [11]. In a study of acutely injured patients, only 84.4% of deltoid ligament injuries were diagnosed on arthroscopy [28], with an inability to assess the superficial deltoid ligament bands [78].

### Treatment

#### Nonoperative

In general, the nonoperative management of medial ankle ligament injuries is comparable to injuries of the lateral ankle and includes cessation of activities, ice, anti‐inflammatories and physiotherapy with or without medial ankle support for the majority of those with Type I or II ligamentous injuries (partial tear or incomplete tear with only mild functional impairment) [14, 40, 78]. Physiotherapy may focus on muscular strengthening, proprioception rehabilitation [17] or neuromuscular rehabilitation [64].

Some authors advocate the use of splints, such as Aircast boots (DJO Global) [71] or fibreglass casts (advocated for Type III sprains) [17] or medial arch orthotics [64]. Immobilisation in cast does not appear to contribute to chronic deltoid ligament insufficiency [81].

Good results have been demonstrated in patients with deltoid ligament injuries and associated fibular fractures. In a study of 28 patients with deltoid ligament injuries treated nonoperatively and distal fibular fractures treated with open reduction and internal fixation, 20 patients had a Weber score of 0–2 (Very good—Good), and 8 had a Weber score of 3 (Poor) at 18 months [99]. In a study of 150 patients treated in the same way, 90% had good satisfaction at a mean follow‐up of 3.5 years [12]. Those patients who were unsatisfied were found to be from a group with high‐energy injuries. A study of 50 patients with associated fibular fracture requiring open reduction internal fixation was randomised into nonoperative or suture fixation of the deltoid ligament. At a mean follow‐up of 17 months, there was no significant difference in working ability, sports activity, pain or swelling [81]. Other studies have echoed these findings [22, 73, 80, 99].

Only a few studies have reported conflicting results. In an observational prospective cohort study of 30 patients with fibula fractures treated with open reduction internal fixation and evidence of deltoid ligament injury on stress views, 41% of patients still experienced pain while walking and 34% had activity limitation due to pain at 15 months post‐op. Although ankle instability was present in 17% of cases, this was rarely determined to be the cause of the pain, and it was felt that of those with persisting pain, chronic over‐use of an attenuated deltoid ligament was responsible in 34% of patients [37].

We believe that patients should be evaluated with stress imaging, and the majority of patients should be treated with a trial of nonoperative treatment. If instability is identified, surgery should be considered on an individual basis.

### Surgical treatment

#### Open deltoid ligament repair

Although there have been no randomised control trials, a meta‐analysis performed in 2019 compared outcomes in patients who had suffered ankle fractures with associated deltoid ligament injury. Three comparative studies were included with a total of 192 patients who had undergone either nonoperative management of the deltoid ligament or primary suture repair. Those who were treated with deltoid ligament repair had superior MCS with better pain scores at final follow‐up, but no difference in functional outcomes or complication rates [76].

The surgical technique of open deltoid ligament repair depends on the location and severity of the injury. In the paper by Hintermann et al [27]., the steps are as follows:

- 4–8 cm curved incision starting 1–2 cm proximally to the tip of the medial malleolus and curved anteriorly.
- The fascia is incised exposing the deltoid ligament and the posterior tibial tendons.
- Type I Injuries: The anterior aspect of the medial malleolus is exposed and freshened, and a suture anchor is placed 4–6 mm above the tip of the medial malleolus. The detached ligament is taken by the suture and the interval is closed.
- Type II Injuries: The damaged deltoid ligament is divided into two flaps. The deep aspect is fixed to the medial malleolus in the same fashion as Type I injuries. The superficial aspect of the deltoid ligament is fixed to the superior navicular tuberosity using a bone anchor.
- Type III Injuries: The detached deltoid and spring ligaments are attached to the navicular tuberosity using bone anchors.

#### Arthroscopic repair

Some surgeons have advocated arthroscopic repair, which has been advocated in patients at risk of complications of open surgery [41]. The purported benefit of arthroscopic treatment is that it allows for evaluation of the articular surface, and debridement of the deltoid ligament preventing adequate reduction of the bony mortise in those with fibular fractures.

The technique is described as follows:

- An anteromedial approach to the ankle is utilised.
- Two anchors are placed in the anterior aspect of the medial malleolus.
- Anchor sutures exit the portal, the sutures are passed through the deltoid ligament using a suture passer, exiting through the skin.
- Fibular fractures are reduced and stabilised.
- The deltoid ligament repair is tensioned with the ankle in neutral dorsiflexion and slight inversion.

As mentioned in the imaging section, arthroscopic techniques are not appropriate for repairing superficial deltoid ligament injuries in cases where the deep ligament injuries are intact [78].

A report on combined all‐arthroscopic medial and lateral ligament repair demonstrated good outcomes with a median American Orthopedic Foot & Ankle Society (AOFAS) score increasing from 70 to 100 at the final follow‐up [89]. This study did not have a comparator, so we are unable to comment on whether these patients did better or worse than patients being treated nonoperatively or with open ligament repair. A retrospective case series published in 2020 of 20 ankles with arthroscopic repair of the deltoid ligament produced similar results, albeit without a comparator group [3].

A later review of 388 patients suffering deltoid ligament injury in conjunction with a Weber B or C ankle fracture and comparing repair versus nonrepair suggested better radiographic findings, AOFAS score and rate of complication when the deltoid ligament was fixed at the same time as the ankle fracture ORIF [21].

#### Deltoid ligament reconstruction in acute injury

Deltoid ligament reconstruction is not advocated in acute cases but may be required in particularly severe trauma. A case study reported on the use of a plantaris tendon autograft to reconstruct the deltoid ligament in an injury involving grinding of the medial malleolus. In this case, the ankle was grossly unstable with overlying skin loss. At the 30‐month follow‐up, the patient had returned to sporting activities and was completely asymptomatic [6]. A further case report has demonstrated the use of the posterior tibial tendon as an autograft in a patient with established flatfoot deformity [68].

### Treatment decisions

#### Should the deltoid ligament be repaired in ankle fractures?

Ankle fractures with deltoid ligament injuries are often unstable, and open reduction and internal fixation of the fibula fracture is therefore recommended. Fibula fixation is well accepted, but deltoid ligament repair is controversial. Many studies suggest that acute repair is unnecessary [2, 22, 53, 81, 84, 87, 99]. Although these studies use a comparator group, none are randomised or controlled in any way.

Other studies recommend fluoroscopic stress assessment of the deltoid ligament with an assessment of the syndesmosis [5, 31, 51, 90, 91]. These authors feel that if the ankle mortise remains unstable once bony reduction and stabilisation have been achieved, then soft tissue repair should be performed. A retrospective study of 78 patients treated with open reduction internal fixation of the fibula with or without deltoid ligament repair found favourable outcomes in those treated with deltoid repair. This study also found that in the case of both deltoid ligament and syndesmotic instability, a deltoid ligament repair was enough to restore the stability of the ankle joint [96].

It should be noted that medial malleolar fractures can occur in conjunction with deltoid ligament injuries; in those cases, bony fixation of the medial malleolus will not address the ligamentous instability [78]. A study of 27 patients demonstrated that 26% of patients with a stabilised medial malleolar fracture had ongoing radiographic evidence of deltoid ligament incompetence [86].

A comparative study of medial malleolar fractures with associated deltoid ligament injury treated with either standard open reduction internal fixation with deltoid ligament repair and trans‐articular external fixation found equivalent outcomes in the two groups with the deltoid ligament repair group having earlier functional recovery [47].

Another study has demonstrated that although patient outcomes were not significantly different, surgical repair of the deltoid ligament helped reduce the MCS and mal‐reduction rate, especially in patients with Weber C ankle fractures [91].

A retrospective review of 108 patients with isolated unstable distal fibula fractures compared the outcomes of patients who underwent (1) ORIF + deltoid repair versus (2) ORIF + transyndesmosis fixation versus (3) ORIF + deltoid repair and transdyndesmosis fixation. The authors found no difference in American Academy of Orthopedic Surgeons Lower Limb Outcomes Assessment: Foot and Ankle Module (AAOS‐FAM) scores. The reoperation rates were 0% in the deltoid repair group, 26% in the trans‐syndesmosis repair group and 23% in the combined fixation group. Reoperations were mainly for the removal of previously implanted hardware. The authors conclude that direct deltoid ligament repair may be a favourable strategy compared with trans‐sydesmosis fixation in unstable distal fibula fractures [94].

The patient population also needs to be considered, as athletes have different requirements to those suffering low‐energy insufficiency injuries. A case series of 14 National Football League (NFL) players with superficial deltoid complex avulsion injuries during high‐energy ankle fractures were treated with open reduction and internal fixation of the fibula fracture and open deltoid repair. All patients had a return to activity 6 months after injury with no difference in pre‐ and postsurgery playing experience. A total of 86% of the patients had a return to competitive play [33].

The aims of treating ankle fractures are to reduce the ankle mortise and stabilise unstable injuries. There are no long‐term follow‐ups of patients who have had repair of the deltoid ligament in this patient group, and studies are generally low‐powered.

### Rehabilitation

In nonoperative cases, support with a nonweight‐bearing plaster cast or supportive boot for 3–4 weeks followed by gradual physiotherapy is recommended until the ankle is asymptomatic [34].

Although rehabilitation in operative cases is dependent on joint stability [75], in general, the ankle requires 6 weeks of nonweight bearing in a cast. After this time, protected and progressive weight bearing in a removable boot is used for a further 4–6 weeks with progressive physiotherapy [50].

## DISCUSSION

The most important finding of the present study was that acute repair or reconstruction of isolated deltoid ligament injuries should be considered for patients presenting with ankle instability. Acute ankle injuries represent a significant burden of disease in the United Kingdom [7], representing up to 30% of all sporting injuries [18, 48, 93]. Deltoid ligament injuries are a subset of ankle sprains, often found in conjunction with other ligament or bony injuries. Neglected deltoid ligament injuries can lead to progressive deformity, posterior tibial tendon dysfunction, flat‐foot deformity and a valgus hindfoot. Some studies have reported on patients having persistent medial gutter pain and medial ankle instability after an isolated deltoid ligament injury, raising the question of whether they should have had acute primary repair [22, 37, 81, 83, 99]. Furthermore, a 20.4% incidence of posttraumatic ankle osteoarthritis has been reported in patients with untreated isolated deltoid ligament injury [31].

A large multicentre study of 1533 ankle fractures identified 131 patients with deltoid ligament injury who underwent primary repair and suggested that those treated with repair had improved postoperative medical clear space, ankle instability, with improved AOFAS, Visual Acuity Scale (VAS) and SF‐36 scores [97]. This was a level III study, as there was no randomisation of patients found with a deltoid ligament injury into treatment arms.

The vast majority of patients with isolated deltoid ligament injuries will not require surgical intervention and can be treated with immobilisation and activity cessation with progressive physiotherapy. Reconstruction in the acute setting should be considered for those with evidence of ankle mortise instability on clinical assessment and/or stress imaging.

Deltoid ligament injury is a common finding in patients with acute ankle fractures. An arthroscopic study of 288 patients revealed medial ligament injury in 39.6% of cases [28]. The decision to operate on a fibular fracture is dependent on the fracture pattern, patient factors and instability [52]. If a fibular fracture requires surgery, then this should be stabilised prior to the assessment of the stability of the ankle mortise. If the mortise remains unstable despite fibular fixation, then deltoid ligament repair should be considered.

Medial instability can be due to deltoid ligament injury, medial malleolar fracture or a combination of the two. Two studies have determined that patients with medial instability due to medial malleolar fractures had worse outcomes than those with deltoid ligament injuries [82, 85]. Better outcomes were seen in the group with lateral malleolus and deltoid ligament injury, thought to be due to differing injury patterns and energy expended [28].

With regard to the role of syndesmosis with deltoid ligament injury in ankle stability, studies have found that equivalent outcomes were found in groups where either the syndesmosis or the deltoid ligament injury was repaired [38]. This raises the question of whether both the deltoid ligament and syndesmosis require fixation in these cases.

## CONCLUSIONS

There is currently no consensus on the treatment of deltoid ligament repair in the acute setting, and there is a lack of high‐powered evidence to support any recommendations. Furthermore, complications do not occur for a number of years and as such, long‐term follow‐up is required to assess for ongoing medial ankle instability, pain, dysfunction and deformity.

## AUTHOR CONTRIBUTIONS

Jacob Koris and Arul Ramasamy performed the initial review and drafted the paper. James D. F. Calder, Miki Dalmau‐Pastor and Miguel A. Fernandez significantly contributed to redrafting and finalising the paper.

## CONFLICT OF INTEREST STATEMENT

The authors declare no conflicts of interest.

## ETHICS STATEMENT

Ethical approval was not sought for the present study because it did not directly involve human participants. Informed consent was not sought for the present study because it was an analysis of existing published work. All authors consent to publication.

## Data Availability

This report does not contain patient‐identifiable data.
